# The Chemokine Receptor CXCR4 Strongly Promotes Neuroblastoma Primary Tumour and Metastatic Growth, but not Invasion

**DOI:** 10.1371/journal.pone.0001016

**Published:** 2007-10-10

**Authors:** Roland Meier, Annick Mühlethaler-Mottet, Marjorie Flahaut, Aurélie Coulon, Carlo Fusco, Fawzia Louache, Katya Auderset, Katia Balmas Bourloud, Estelle Daudigeos, Curzio Ruegg, Gilles Vassal, Nicole Gross, Jean-Marc Joseph

**Affiliations:** 1 Department of Paediatrics, Paediatric Oncology Research, University Hospital Centre Hospitalier Universitaire Vaudois Lausanne (CHUV), Lausanne, Switzerland; 2 Division of Experimental Pathology, University Institute of Pathology, Lausanne, Switzerland; 3 INSERM U362, Institut Gustave Roussy, Villejuif, France; 4 Unité Propre de Recherche de l'Enseignement Supérieur EA3535, Institut Gustave Roussy, Villejuif, France; 5 Division of Experimental Oncology, Multidisciplinary Oncology Centre (CePO), Lausanne Cancer Centre, Lausanne, Switzerland; 6 Department of Paediatrics, University Hospital Centre Hospitalier Universitaire Vaudois Lausanne (CHUV) Surgery Unit, Lausanne, Switzerland; Canadian Agency for Drugs and Technologies in Health, Canada

## Abstract

Neuroblastoma (NB) is a heterogeneous, and particularly malignant childhood neoplasm in its higher stages, with a propensity to form metastasis in selected organs, in particular liver and bone marrow, and for which there is still no efficient treatment available beyond surgery. Recent evidence indicates that the CXCR4/CXCL12 chemokine/receptor axis may be involved in promoting NB invasion and metastasis. In this study, we explored the potential role of CXCR4 in the malignant behaviour of NB, using a combination of *in vitro* functional analyses and *in vivo* growth and metastasis assessment in an orthotopic NB mouse model. We show here that CXCR4 overexpression in non-metastatic CXCR4-negative NB cells IGR-NB8 and in moderately metastatic, CXCR4 expressing NB cells IGR-N91, strongly increased tumour growth of primary tumours and liver metastases, without altering the frequency or the pattern of metastasis. Moreover shRNA-mediated knock-down experiments confirmed our observations by showing that silencing CXCR4 in NB cells impairs *in vitro* and almost abrogates *in vivo* growth. High levels of CXCL12 were detected in the mouse adrenal gland (the primary tumour site), and in the liver suggesting a paracrine effect of host-derived CXCL12 on NB growth. In conclusion, this study reveals a yet unreported NB-specific predominant growth and survival-promoting role of CXCR4, which warrants a critical reconsideration of the role of CXCR4 in the malignant behaviour of NB and other cancers.

## Introduction

Neuroblastoma (NB), the most common and deadly solid tumour in childhood, originates from primitive cells of the sympathetic nervous system. NB displays a very heterogeneous behaviour at clinical, biological, and genetic levels. The majority of patients presents at diagnosis with an aggressive and metastatic disease for which little improvement in therapeutic options has been made in the last decade [1]. Metastatic tumour cell dissemination of advanced stage and highly malignant NB tumours mainly occurs in the bone marrow, bone, liver, and skin. Organ-specific metastasis, known to involve events in both the tumour cells and the target tissue, represents an intriguing and yet unresolved phenomenon [2], [3]. Chemokines are small, cytokine-like proteins forming a large superfamily, originally discovered as essential mediators of leukocyte chemoattraction in inflammation and immune cell homing and recirculation. Upon binding to their cognate seven-transmembrane spanning G-protein-coupled receptors, they induce integrin activation and cytoskeletal rearrangement promoting cell adhesion and directional migration [4]–[6]. Emerging evidence indicates that the chemokines and their receptors play an important role in tumour biology [7], [8]. CXCR4 is the chemokine receptor most commonly found on tumour cells. It has been reported expressed in at least 23 different types of cancers [9]. In a number of cancer types, binding of its cognate ligand CXCL12 [10] was reported to mediate directed migration of cancer cells to sites of metastasis [9], [11]. Recent reports indicate that CXCR4 is commonly expressed on NB metastasis in the bone marrow and that it may be actively contributing to NB tumour cell homing to the bone marrow [12]. However, these observations are still controversially discussed [13].

There is emerging evidence that the tumour environment plays a critical role in modulating the behaviour of primary or metastatic tumour cells. The appropriate microenvironment and vascular niche involved in metastasis initiation can only be provided by the orthotopic site [14]. Hence, the use of *in vivo* orthotopic models, faithfully reproducing the tumour microenvironment, is essential for the analysis of metastasis-related mechanisms [15]–[18].

In this study, we investigated the contribution of CXCR4 to NB progression *in vitro* and *in vivo* and demonstrate that CXCR4 overexpression promotes NB primary and secondary tumour growth but not NB invasion. Using an *in vivo* orthotopic model [19], we demonstrate that CXCR4 has no significant influence on the number or pattern of NB metastasis. In contrast, the strong growth-promoting effect observed may represent the main role of CXCR4 in tumour progression. These results warrant a critical reconsideration of the role of CXCR4 in NB tumour progression.

## Materials and Methods

### Cell lines and culture

IGR-NB8 cell line was derived from a xenotransplanted human stage 3 abdominal NB [18]. IGR-N91 cell line was derived from a stage 4 NB infiltrated bone marrow [20]. The cell line SH-SY5Y was used as a CXCR4 positive control [21]. The prostate cancer cell line PC3, represents another positive control for its described and generally accepted properties upon CXCR4 overexpression [22]. Unless specified, cells were cultured in Dubelcco's modified Eagle's medium (DMEM) supplemented with 2 mmol/l L-glutamine, 10 µg/ml gentamycin, and 10% fetal calf serum (FCS) (AMIMED). For growth and invasion assays, cells were cultured in N2-supplemented DMEM, a serum-free medium specifically defined to support the growth of neuronal cells [22], [23]

### CXCR4 overexpression

A pMIGR-EGFP vector encoding for the enhanced green fluorescent protein (EGFP) with or without the chemokine receptor CXCR4 gene was inserted by retroviral mediated infection into IGR-NB8, IGR-N91 and PC3 cells as previously described [19]. The CXCR4 containing plasmid was sequenced to verify its integrity [12].

GFP-overexpressing cells were sorted by fluorescence activated cell sorting (FACS) and cloned. Control GFP and selected CXCR4 clones were analysed by FACScan (Becton Dickinson), using the PE–labelled 12G5 monoclonal antibody from BD Pharmingen to measure and quantify CXCR4 surface expression levels.

### In vivo growth

Animal engraftments, tumour and metastases growth records were performed as previously described [19]. Athymic locally bred Swiss nude mice were engrafted with 6×10^5^ of NB8-E6 (8 animals), NB8-CXCR4-C3 (8 animals), N91-E2 (8 animals), and N91-CXCR4-14 (13 animals) cells in 15 µl DMEM in the left adrenal gland. For knock-down experiments, 2×10^6^ N91-pAB303, N91-shCXCR4-CS1 and N91-shCXCR4-CS2 cells in 50 µl DMEM were injected. Ten animals were used for each group. One mouse in the N91-pAB303, 4 in the N91-shCXCR4-CS1 and 2 in the N91-shCXCR4-CS2 group died of perioperative complications.

Micrometastases were detected by PCR and macroscopic metastases by morphological examination and/or GFP-IHC as previously described [19].

### Cell proliferation assay

Cell proliferation was assessed using the MTS/PMS cell proliferation kit from Promega as described earlier [24]. Twenty thousand cells per well were seeded in either 10% FCS or 2% FCS. Where indicated, growth assays were performed in the presence of CXCL12 (100 ng/ml).

### ERK1/2 phosphorylation

As described [25], cells were serum-starved overnight and washed once with PBS prior to experimentation. Aliquots of 10^6^ cells were stimulated with CXCL12 (Preprotech) for indicated time. Incubations were stopped by the addition of 500 µl SDS-PAGE buffer. To detect phospho-ERK1/2, membranes were first probed with a phospho-specific antibody (Cell Signalling) and then stripped and re-probed with the antibody against the total ERK1/2.

### CXCL12 ELISA

An ELISA assay (R&D Systems) was used to quantify the production of CXCL12 by NB cell lines, normal mice tissues, and orthotopically grown primary tumours. Tissue extracts were prepared as described [26] and were processed following R&D Systems's instructions.

### Chemotaxis and invasion assays

Cell migration was measured using Transwell Costar® cell culture chambers with polycarbonate filters of 8 µM porosity. Briefly, 2.5×10^5^ cells suspended in serum-free medium (SFM) were seeded in the upper compartment of a two chambers system, as described [27]. The lower compartment was filled with SFM with or without 100 ng/ml CXCL12. Migration assays were performed in the presence or absence of a specific CXCR4 receptor inhibitor, 4F-benzoyl-TN14003 [28]. The cells were allowed to settle down for 4 hours. After washing, membranes were fixed with 4% paraformaldehyde in PBS for 10 min and stained with haematoxylin. Non migrated cells were scraped from the upper side of the filter, and migrated cells on the lower side counted by light microscopy.

For invasion assays, the 8 µm polycarbonate membranes of the Transwell inserts were coated with 50 µl of 0.8 mg/ml of Matrigel®. The lower chamber was either supplemented with DMEM-N2 medium alone or with DMEM-N2-containing 100 ng/ml CXCL12. Fifty thousand overnight serum-starved cells were plated in the upper chamber. After 48 h incubation at 37°C, membranes were processed and number of invading cells counted as above.

### Knock-down of CXCR4 by shRNA

Stable downregulation of CXCR4 was achieved by RNA interference using short hairpin RNAs [29]. A published sequence from shRNA which specifically and efficiently down regulates CXCR4 was used as previously reported [30], [31]. The sequences were checked by sequencing a PCR-amplified region containing the oligonucleotide. The H1 promoter and the shRNA cassette were sub-cloned into the BamHI/SalI sites of the lentiviral vector pAB286 containing the SV40 promoter-puromycin acetyltransferase cassette [32]. The pAB303 lentiviral vector which contains the H1 promoter alone, without shRNA sequence, was used as a negative control (gift from R. Iggo, Aberdeen, Scotland) [32]. Lentiviruses were produced by co-transducing the lentiviral vector plasmid containing the shRNA (10 µg) and the second generation packaging plasmids (3.5 µg pMD2-VSVG, 6.5 µg pCMVΔR8.91) into 293T (ATCC CRL-11268) all vectors but pAB303 kindly provided from D. Trono, EPFL, Lausanne, Switzerland. Infection of target cells were done by over-night incubation at 37°C in virus-containing media in presence of 8 µg/ml polybrene (hexadimethrindibromide, Fluka). Twenty-four hours post-transfection, the lentivirus containing supernatant was used to infect the IGR-N91 cell line. Selection was started 48 h after infection with 5 µg/ml puromycin (Sigma-Aldrich, Steinheim, Germany). Puromycin resistant infected cells were selected 24 h post-infection.

CXCR4 protein expression knock-down in shRNA-transduced cells was determined by FACS analyses.

### Statistical analyses

Statistcial analyses were performed using GraphPadPrism 4.0. Two-way ANOVA with Bonferroni post-test corrections was performed to compare cell growth in 10% and 2% FCS. Student's t test was performed for cell growth in the presence or absence of CXCL12. Fisher's exact test was performed to compare the number of metastasis-bearing animals. Mann-Whitney test was used to compare *in vivo* tumour growth. A p<0.05 was considered to represent signficance and p<0.01 was considered to be highly significant.

## Results

### Expression of the chemokine receptor CXCR4 on NB cell lines

**Figure 1:**
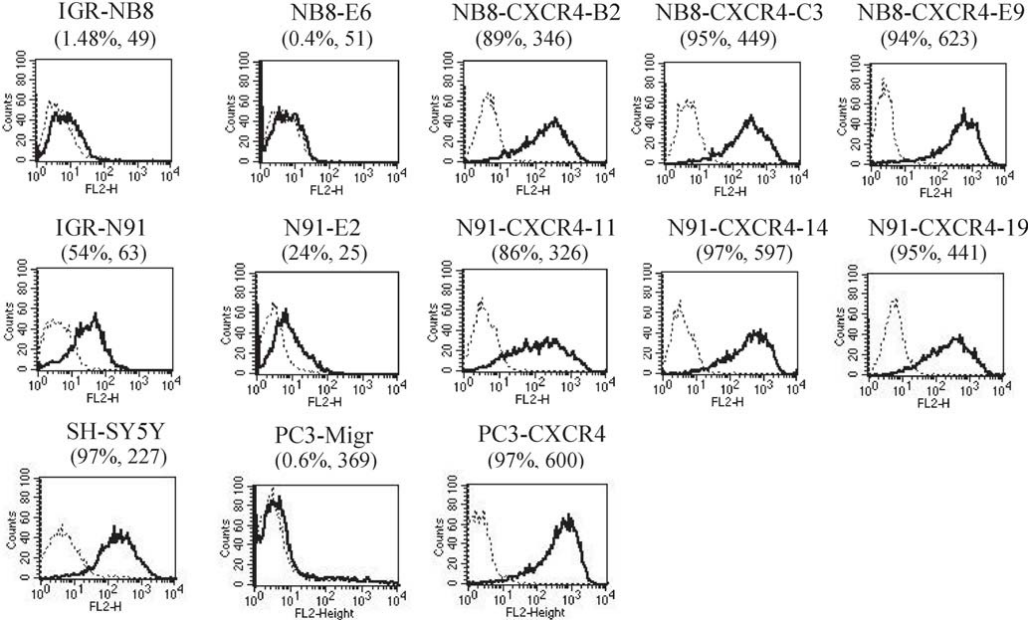
CXCR4 cell surface expression. IGR-NB8 and IGR-N91, two well characterized NB cell lines, and transduced cell lines/clones were analyzed for their cell surface CXCR4 expression by flow cytometry (FACS). Percent positive cells is indicated as well as the mean fluorescent intensity (brackets) of the CXCR4 staining. The NB cell line SH-SY5Y constitutively expressed high levels of CXCR4 as previously described [21]. The prostate cancer cell line PC3 showed comparable levels of CXCR4 expression upon transduction as NB8-CXCR4 and N91-CXCR4 clones.

Constitutive cell surface CXCR4 expression, measured by flow cytometry (FC) in a panel of NB cell lines was variable among the cell lines tested (not shown). As shown in Figure 1, two selected invasive NB cell lines, IGR-N91, SH-SY5Y, displayed constitutive levels of CXCR4 surface protein expression (54% and 97% positive cells respectively), while the non-invasive IGR-NB8 cell line expressed very little if any surface CXCR4 (1.48% positive cells).

To explore the putative role of CXCR4 in NB malignant behaviour, we stably overexpressed CXCR4 by retroviral transduction in two NB cell lines with well-characterized growth properties: IGR-NB8, a non metastatic line, and IGR-N91, which forms metastases when implanted orthotopically [19]. CXCR4-transduced clones, randomly selected from IGR-NB8 (NB8-CXCR4-B2, C3, and -E9) or IGR-N91 (N91-CXCR4-11, -14, -19), displayed high and similar levels of surface CXCR4 expression. Mock transduced NB8-E6 cells remained CXCR4 negative, while and N91-E2 cells express slightly lower levels of CXCR4 compared to parent cells. (Figure 1).

### CXCR4 increases IGR-NB8 in vivo growth

To evaluate the role of CXCR4 in the development of site-specific metastasis we used the orthotopic NB mouse model that strongly implies the role of the tumour microenvironment on tumour progression. Parental lines and CXCR4 transduced clones were analysed *in vivo* for primary (orthoptopic) tumour growth, metastatic dissemination, and organ specific secondary growth.

**Figure 2:**
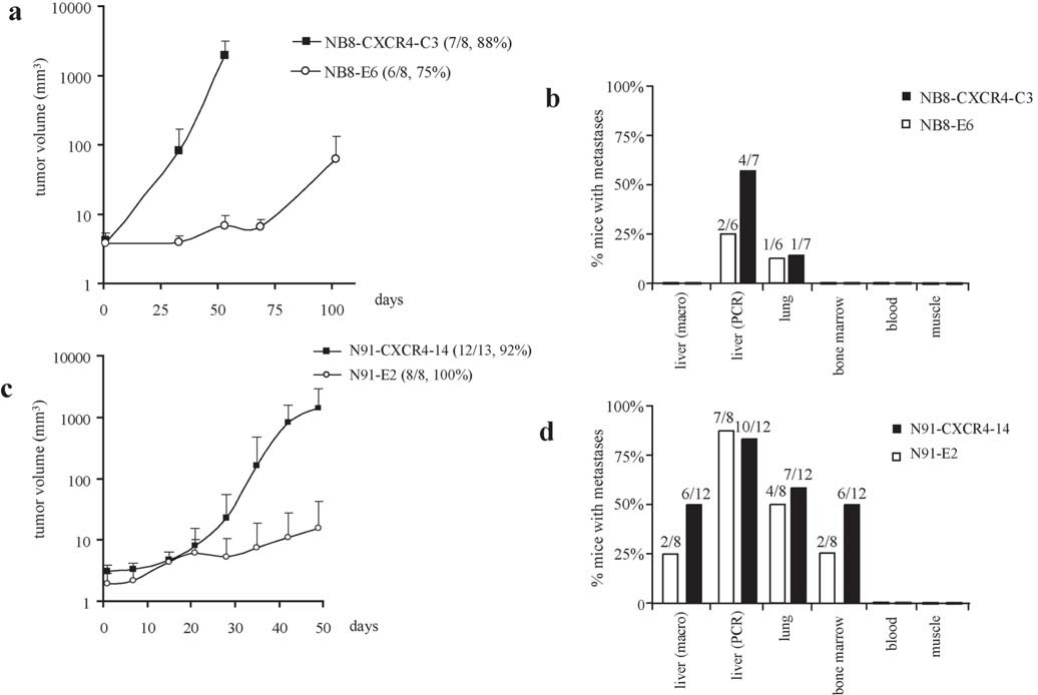
*In vivo* orthotopic tumour growth and metastasis. Tumour growth measured in mice engrafted with NB8-E6 and NB8-CXCR4-C3 (a), and N91-E2 and N91-CXCR4-14 (c) The mean tumour volume ± S.D. as measured by echo-doppler at indicated days after implantation is shown. The tumour take expressed as number of mice with tumour/total mice and percent mice with tumours is indicated. (b) and (d) Detection of metastases in mice engrafted with either NB8-E6/NB8-CXCR4-C3 or N91-E2/N91-CXCR4-14 cells. Macroscopic liver metastases were detected by gross examination. Micrometastases were detected in lung, bone marrow, muscle and blood by GFP-PCR. Bars represent percentages of mice with macroscopic liver metastases or GFP-PCR positive signals in indicated organs per tumour bearing animal.

*In vivo* experiments were first performed with the NB8-E6 cells, previously shown to be able to induce tumours in the orthotopic model, but unable to develop metastases. Two randomly selected control NB8-E6 and CXCR4-overexpressing NB8-CXCR4-C3 cell lines were implanted in the left adrenal gland of nude mice. The mice were examined by echography at day 33, 53, 69, and 102 for the presence of tumours. Six out of 8 animals (75%) in the NB8-E6 group, and 7/8 mice (88%) in the group engrafted with NB8-CXCR4-C3 developed a tumour, thus showing a similar tumour take in mice engrafted with CXCR4-negative or CXCR4 positive NB8 cell line (Figure 2a).

As shown in Figure 2a, CXCR4 expression in the NB8 cell line dramatically accelerated tumour growth. The first echographic record performed at day 33 after implantation, already revealed the presence of tumours with a volume between 100 and 200 mm^3^ in two mice in the NB8-CXCR4-C3 group, whereas no tumours were detected in the control group. At day 53, 7/8 mice in the NB8-CXCR4-C3-implanted group of mice had developed tumours with a mean volume of 1727 mm^3^, which required the immediate sacrifice of all animals in this group, while in the control group tumours were barely detectable at that time point. At sacrifice (day 102), the mean volume of NB8-E6 tumours was only 48 mm^3^. These results reveal that the time interval between engraftment and detectable tumour growth was considerably reduced in mice engrafted with NB8-CXCR4-C3 cells compared to NB8-E6, thus demonstrating that CXCR4 expression in the CXCR4-negative cell line NB8 dramatically accelerated orthotopic tumour growth *in vivo* (Figure 2a).

### CXCR4 does not induce metastatic dissemination of IGR-NB8 cells

One of the major proposed contribution of CXCR4 to NB progression was the promotion of site-specific metastasis [21]. We therefore investigated whether CXCR4 overexpression in NB tumour cells could influence organ-specific invasive behaviour in our *in vivo* model. Major target and control organs of tumour-bearing mice were macroscopically analysed for the presence of metastases. No macroscopic metastasis was detected in either group. Micrometastases as detected by GFP-PCR-based analysis, revealed GFP-signals in the lungs of a minority of NB8-E6 and NB8-CXCR4-C3-engrafted animals, and in the livers of 2/6 NB8-E6 and 4/7 NB8-CXCR4-C3 bearing animals, respectively. No signal was found in blood, muscle, and bone marrow of both groups of mice (Figure 2b). All mice livers were further investigated by GFP-immuno-histochemistry where no microscopic metastasis was detected (data not shown).

These results indicated that enhanced CXCR4 expression in the IGR-NB8 cells tremendously accelerated the growth of the primary tumour. In contrast, CXCR4 overexpression was not sufficient to induce metastatic dissemination in this orthotopic model.

### CXCR4 increases IGR-N91 in vivo growth

Since the IGR-NB8 cell line is a non-metastatic cell-line we were concerned that it may lack essential invasive elements, thereby masking a potential metastasis-promoting effect of CXCR4. We therefore repeated the experiment with IGR-N91, a cell line which is capable of forming metastases when injected orthotopically, but not when injected s.c. [19]. Specifically, we injected the CXCR4 overexpressing cell line N91-CXCR4-14 and N91-E2, the mock-transduced control. Out of 13 mice implanted with N91-CXCR4-14 cells, 12 developed a tumour, with a mean volume of 1390 mm^3^ at 49 days, whereas in the control group, 8/8 developed a tumour, reaching a mean volume of 15 mm^3^ after 49 days (Figure 2c). As observed with the IGR-NB8 derived cells, the tumour take was similar in both groups. N91-CXCR4 tumours also grew considerably faster, reaching nearly 100 fold larger volumes than N91-E2 tumours.

### CXCR4 does not enhance metastatic dissemination of IGR-N91 cells

The examination of the metastatic pattern of these tumours revealed that 6/12 mice in the N91-CXCR4-14 group had at least one macroscopic liver metastasis, whereas in the control group 2/8 mice developed macroscopic hepatic metastases (Figure 2d). The volume of liver metastases in N91-CXCR4-engrafted group of mice was much larger (250 to 1000 mm^3^) when compared to the N91-E2 group (less that 100 mm^3^). GFP-PCR analyses revealed the presence of micrometastases in 58% of the lungs and 50% in the bone marrow of mice with N91-CXCR4-14 tumours, versus 50% and 25% in the N91-E2 group. These differences observed in the occurrence of micro- and macro-metastases were nevertheless not statistically significant (Fisher's exact test: p = 0.38), while the differences in the volume of macroscopic liver metastases in the two groups were highly significant. No GFP-PCR signal was detected in the blood or in the control organ (muscle) in either group.

These results reveal that CXCR4 overexpression in the metastasis-competent IGR-91 NB cells has a strong and predominant effect on primary and secondary tumour growth (increase of the metastases volume). Even in such orthotopic model, a statistically significant increase of metastases frequency could not be demonstrated.

### Functional CXCR4 supports in vitro NB cell growth and survival

To gain further insight into the distinct molecular mechanisms responsible for the CXCR4/CXCL12 axis mediated effects on NB progression rather than site-specific invasion, we next explored the effects of CXCR4 over-expression on *in vitro* proliferation, survival, migration, and invasion of these NB cells.

**Figure 3:**
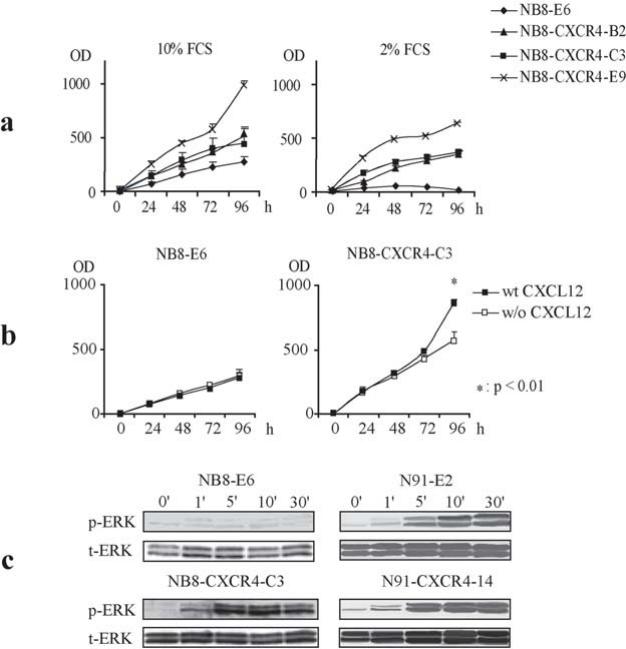
Effect of functional CXCR4 overexpression in NB cell lines. (a) I*n vitro* growth of NB8-E6, NB8-CXCR4-C3, -B2, and -E9 cells, in 10% (black squares) or 2% serum (open squares). (statistical analysis by two-way ANOVA and Bonferroni correction) A representative of three independent experiments is shown. (b) *In vitro* growth of NB8-E6 and NB8-CXCR4-C3 cells in the presence of 100 ng/ml CXCL12 (black squares) or in medium alone (open squares). (c) Western blot analysis of ERK1/2 phosphorylation in response to CXCL12 in CXCR4-transduced cells.

The growth and survival of different CXCR4-expressing cell lines was measured *in vitro* in complete medium (10% FCS) or in stress conditions (2% FCS) (Figure 3a). Growth curves were established over a 96 hours period. In the presence of 10% FCS, the results revealed a non significant difference in the growth capacity of 2 NB8-CXCR4-expressing clones as compared to NB8-E6. A significantly enhanced growth of one clone (NB8-CXCR4-E9) as compared to the control cells was observed. (Figure 3a). Thus *in vitro*, the IGR-NB8-E6 and NB8-CXCR4-C3 cells selected for in vivo studies, did not show different growth properties. In contrast, addition of the CXCL12 ligand significantly increased the growth of CXCR4-overexpressing NB8-CXCR4-C3 cells, but not that of the control NB8-E6 cells (Figure 3b).

The CXCR4-expressing clones and their controls were exposed to suboptimal culture conditions (2% FCS). These stress conditions significantly impaired NB8-E6 growth, while they only slightly affected the growth of the three CXCR4 expressing clones, indicating a significant (p<0.05) CXCR4-mediated enhanced survival in stress conditions (Figure 3b).

CXCR4 overexpression in the IGR-N91 cells did not further increase their *in vitro* growth or survival (not shown).

Extracellular regulated kinases (ERK) 1/2 are activated by phosphorylation in response to various extracellular signals, including the binding of CXCR4 to its unique ligand, CXCL12, [22]. To test the functionality of endogenous and transduced CXCR4 in NB cells, we monitored CXCL12-mediated ERK 1/2 phosphorylation. As shown in Figure 3c, no significant increase in ERK1/2 phosphorylation was detected in the control NB8-E6 cells in response to CXCL12 up to 30 minutes. In contrast, in N91-E2, expressing endogeneous CXCR4 and in NB8-CXCR4-C3, and N91-CXCR4-14 cells with exogeneous CXCR4 overexpression, CXCL12 stimulation increased ERK1/2 phosphorylation already after 1 minute. Total ERK1/2 protein was unchanged at all time points (Figure 3c). Altogether these assays confirmed that the CXCR4 receptor, either endogenous or exogenous, is functional and able to transduce CXCL12-mediated intracellular signals in NB cells, implicating the ERK pathway as potential mediator of CXCR4-induced proliferation.

### CXCR4 induces cell migration in response to CXCL12

**Figure 4:**
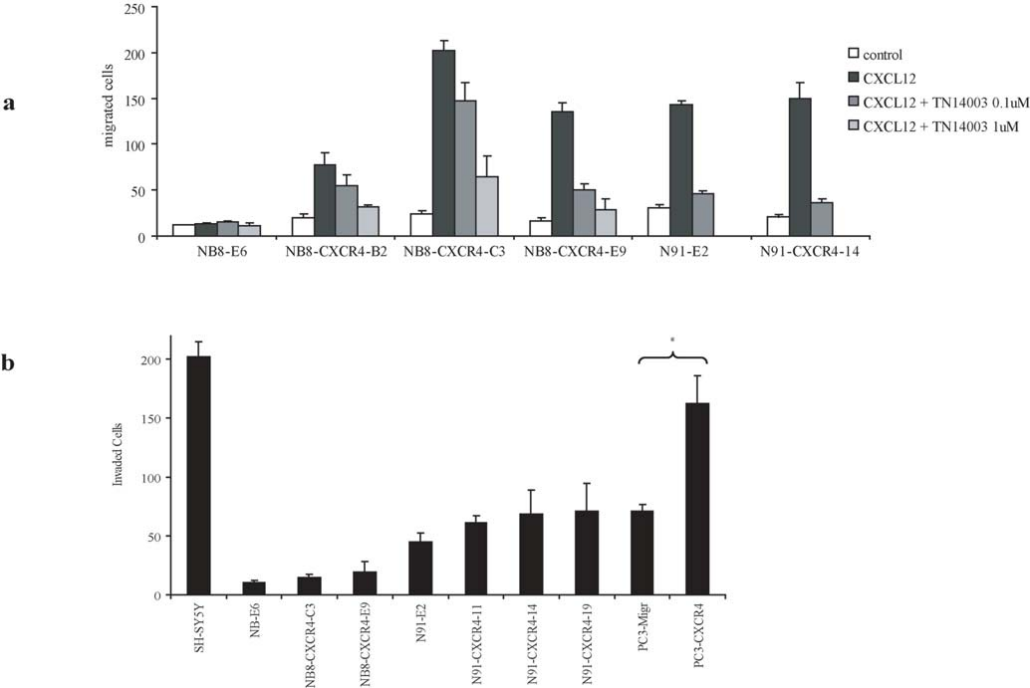
NB8 cell migration and invasion *in vitro*. (a) Migration of NB8-E6, N91-E2, and CXCR4-overexpressing clones (NB8-CXCR4-C3, -B2, -E9, N91-CXCR4-14) towards CXCL12, in absence or presence of the CXCR4 blocker (TN14003 inhibitor). Results of a representative experiment are shown and are expressed as mean number of cells in one field. Five fields were counted for each result. The experiment was performed in triplicates and repeated three times. Error bars indicate S.D. (b) Invasion of SH-SY5Y, NB8 and N91 control and CXCR4 transduced clones through Matrigel®-coated membranes in N2 supplemented serum-free medium alone, with or without CXCL12. CXCL12-mediated invading cells (invaded cell in the absence of CXCL12 have been subtracted) -in a typical field are shown. The mean number of invading cells in 5 fields is given. Error bars indicate S.D. (* indicates p<0.05, Student's t-test)

We next tested whether CXCR4 increased directed migration. To this purpose, CXCL12-mediated cell migration was measured in NB cells. Αs shown in Figure 4a, none of the cell lines were able to migrate in the absence of the ligand. In the presence of 100 ng/ml of CXCL12, migration was induced in IGR-N91 cells and in all three NB8-CXCR4 clones but not in the CXCR4-negative NB8-E6 cells. Migration of N91-CXCR4-14 clone was not enhanced as compared to that of N91-E2 control cells in response to CXCL12.

Migration assays were then performed in the presence of CXCL12 ± the CXCR4 TN14003 inhibitor. TN14003 did not alter the migration of the control NB8-E6 cell line, but it attenuated chemotaxis of CXCR4-expressing clones in a dose-dependent manner (Figure 4a), indicating a specific CXCR4-promoting chemotaxis in IGR-NB8 and IGR-N91 cells.

### CXCR4 does not mediate in vitro invasion of NB cells in response to CXCL12

In order to form metastases *in vivo*, malignant cells need not only to migrate, but also to invade basal membranes and the surrounding tissue. To investigate the role of CXCR4/CXCL12 axis in promoting cell invasion we performed an *in vitro* Matrigel® invasion assay. The different cell lines were placed in the upper chamber of a Transwell® filter coated with Matrigel®, and cells were allowed to invade for 48 hours in response to CXCL12 present in the lower compartment [33].

As shown in Figure 4b, none of the cell lines were invasive in serum free, N2-supplemented medium, whereas control cells SH-SY5Y were invasive in these conditions. In the presence of CXCL12, the N91-E2 cells were only slightly invasive, whereas the NB8-E6 cells were not (Figure 4b). As a control, the prostate cancer cell line PC3, reported to be more invasive when overexpressing CXCR4 [22] was transduced with the same CXCR4 containing plasmid. The CXCR4 transduced cells PC3-CXCR4 cells were significantly more invasive *in vitro* than control cells (Figure 4b) (Student's t-test: p<0.05). However, despite high and similar CXCR4 induced surface expression in NB8-CXCR4, N91-CXCR4 and PC3 cells (Figure 1), the invasive capacity of NB8-CXCR4 and N91-CXCR4 cells was not significantly enhanced as compared to control mock-transduced cells, while significant enhanced invasion of CXCR4-PC3 cells was observed. This suggests a tumour type specific effect of CXCR4 in promoting invasion.

As NB8-CXCR4 overexpressing cells displayed enhanced *in vitro* migration but not invasion nor *in vivo* metastatic dissemination, we investigated whether the lack of invasive properties was due to a lack of proteolytic activities in the NB8 or N91 cells by measuring MMP-2 and -9 activities in SH-SY5Y cells culture supernatant, N91-E2, NB8-E6 and NB8-CXCR4 clones using a zymography assay (Figure S1). Data obtained ruled out an implication of MMP-2 and MMP-9 in the lack of metastatic properties of NB8 cell line used (Figure S1).

### The CXCR4 ligand, CXCL12 is produced by the tumour microenvironment

**Figure 5:**
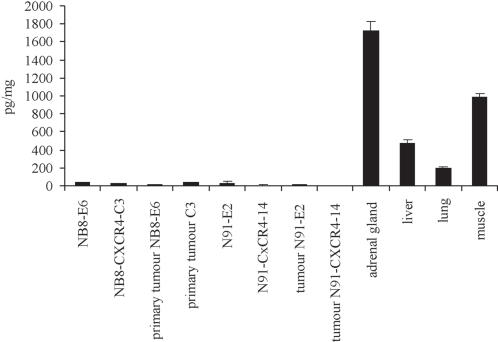
CXCL12 production in mouse tissues and NB tumours. The production of CXCL12 was measured by ELISA in normal mouse tissues, as well as in the different clones, and primary tumours. Results of triplicates are expressed as pg of CXCL12 per mg of extracted protein. One representative of three independent experiment is shown. Experiments were performed in triplicates.

The above data indicate that CXCR4 enhances tumour growth *in vivo* and NB cell proliferation *in vitro* in response to CXCL12. To collect evidence whether CXCL12 may be a factor relevant to tumour growth in vivo, we measured the concentration of CXCL12 in the primary and secondary tumours as well as in selected host tissues (Figure 5). Presence of CXCL12 could be measured by ELISA in several nude mouse organs, and the highest levels of CXCL12 were found in the adrenal gland, the natural primary tumour microenvironment for neuroblastoma. High CXCL12 levels where also found in the liver, lung and muscle, while almost undetectable CXCL12 were measured in bone marrow (not shown) (Figure 5). Surprisingly, no direct relationship was observed between expression levels of CXCL12 in the different organs and the pattern of NB metastasis. In contrast, the high levels of CXCL12 detected in the adrenal glands and liver, may be related to the increased primary NB tumour and secondary metastatic growth in these tissues.

### Knockdown of CXCR4 by shRNA

**Figure 6:**
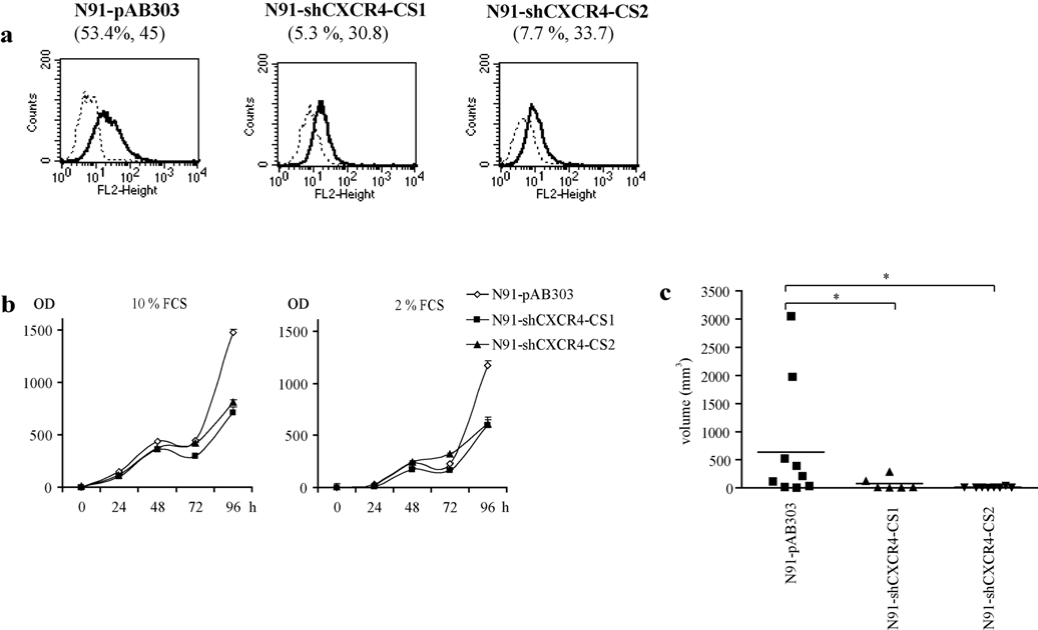
(a) FACS profiles of shRNA-mediated cell surface CXCR4 silencing in N91-shRNA-CS1 and N91-shRNA-CS2 clones and N91-pAB303 control cells. Percent positive cells is indicated as well as the mean fluorescent intensity (brackets) of the CXCR4 staining. (b) *In vitro* growth capacities of N91-shRNA-CS1, N91-shRNA-CS2 clones and N91-pAB303 control cells. (c) *In vivo* growth capacities of IGR-N91 cells were CXCR4 has been knocked down by shRNA. Comparison of tumour volumes after 44 days when mice were sacrificed. * indicates p<0.05 (Mann-Whitney test).

In order to confirm the specific role of CXCR4 in NB, we analysed the growth properties of CXCR4 knock-down cells. Since we observed that in vitro growth, migration and invasion were not further enhanced by exogeneous CXCR4 overexpression in IGR-N91 cells with conctitutive endogeneous CXCR4 expression, we knocked-down endogeneous CXCR4 in IGR-N91 cells. Stable knock-down of CXCR4 was achieved by transduction of IGR-N91 cells with shRNA lentiviral constructs. Clones were screened for efficient silencing of CXCR4 by real-time PCR. CXCR4 mRNA levels of cells transduced with the empty vector pAB303 were used as reference. Resulting cell surface CXCR4 expression, as illustrated in Figure 6a, shows that strong and efficient knock-down of CXCR4 was achieved in both N91-shRNA-CS1 and N91-shRNA-CS2 clones as compared to N91-pAB303 control cells (5.3% and 7.7% vs 53% CXCR4 positive cells respectively).

### Knockdown of CXCR4 impairs in vitro growth

CXCR4 overexpression in NB cells increased *in vitro* cell growth under serum deprived conditions and *in vivo* tumour growth. Cells where CXCR4 has been knocked down by shRNA were tested for their *in vitro* growth capacities. As shown in figure 6b, Both N91-shCXCR4-CS clones revealed impaired *in vitro* growth, not only in serum deprived culture conditions (2% FCS) but also in normal conditions (10% FCS). Interestingly the difference was only apparent at time point 96 h (Fig 6b).

### Knockdown of CXCR4 abrogates in vivo growth

We then specifically investigated the *in vivo* growth capacities of control N91-pAB303, N91-shCXCR4-CS1 and N91-shCXCR4-CS2 cells. For this purpose cells were orthotopically injected in nude mice and tumour growth measured every other week. In this experiment, all mice were sacrificed at day 44 when large tumours were present in the control group (N91-pAB303). As shown in figure 6c, at day 44, N91-pAB303 tumours were significantly larger (mean volume 703 mm^3^) than CXCR4 knocked-down N91-shCXCR4-CS1 and N91-shCXCR4-CS2 tumours (mean volume 74.8 mm^3^ and 9.7 mm^3 ^respectively) (p<0.05 Mann Whitney test).

## Discussion

Recent reports suggest a role for CXCR4 and its ligand CXCL12 in the malignant behaviour of cancer cells. In particular, the CXCR4/CXCL12 axis has been involved in metastasis formation, increased survival of cancer cells under harsh conditions, and establishment of a tumour promoting cytokine/chemokine network [7]–[9], [34]–[38]. The specific involvement of the CXCR4/CXCL12 axis in cell proliferation, and organ-specific metastasis has been partially addressed and remains controversial in some tumour systems, including NB [13]. It has been proposed that CXCR4 expression may be a mechanism by which NB cells metastasise at specific sites [12], [21]. A recent study on NB cell lines and patients samples, however, raised doubts on the implication of CXCR4 in the metastatic dissemination. Even though CXCR4 was expressed on bone marrow metastases, it appeared not to be functional [13].

The present study was initiated to address this controversial topic and to investigate the consequences of CXCR4 expression on growth and metastatic behaviour of two NB cell lines *in vivo*. The question whether CXCR4 is sufficient to initiate and to increase NB metastases upon orthotopic implantation has not been addressed yet. We used a murine orthotopic model of NB which implies the influence of the natural tumour microenvironment on tumour progression and mimics human NB behaviour [19]. The CXCR4/CXCL12 axis-mediated mechanisms were then further investigated *in vitro.*

Our *in vivo* data indicate that almost all mice orthotopically engrafted with NB cells developed a tumour (Figure 2a and 2c). Surprisingly, overexpression of CXCR4 did not lead to induction of metastases in the non-metastatic NB cell line IGR-NB8 and did not increase the frequency of metastases formed by the metastatic IGR-N91 cell line, which constitutively expresses moderate levels of CXCR4 [19] (Figure 2d, Fisher's exact test: p = 0.38). CXCR4-overexpression in both cell lines, however, promoted a CXCL12-dependent chemotaxis, thereby demonstrating that in both cells lines ligand-bound CXCR4 can elicit a motility phenotype. This observation is consistent with previous reports linking CXCR4 expression with tumour cell motility and chemotaxis in response to CXCL12 [5]–[7], [39]–[41]. The controversial data on the influence of CXCR4 on metastasis frequency may be explained by the different in vivo assays used, as well as the different tumour types analysed. [7], [22], [34]–[36], [38]. While neither NB8-CXCR4 nor N91-CXCR4 clones showed any increased capacity to invade Matrigel® in response to CXCL12, CXCR4 overexpressing PC3 prostate cancer cells indeed showed increased in vitro invasive capacity [30]. This observation made in another cancer type confirms previous reports [22] and suggests that the pro-invasive effects of CXCR4 may be tumour type specific.

On the other hand, increased metastatic capacity of CXCR4 expressing NB cells and increased CXCR4 expression in metastatic NB tumour cells as observed by Zhang et al. [12], may be explained by the *i.v*, implantation of tumour cells, thus focussing on late and bypassing the initial steps of metastasis. However, in an orthotopic model of neuroblastoma, which includes all steps of the metastatic process, CXCR4 is not sufficient to induce or increase metastasis, but fully manifest its growth promoting effects.

The reason for the lack of increased invasion and metastasis upon CXCR4 overexpression in NB cells remained elusive. While for IGR-NB8 cells it could be argued that they lack essential invasive or metastatic properties, this cannot be true for IGR-N91 cell lines overexpressing CXCR4, since the parental line has already metastatic capacities. Also, MMP-2 and -9 appear not to be factors limiting invasion and metastatis in these cells. This finding indicate that neither CXCR4 expression nor MMP-2 and –9 are sufficient to confer invasive and metastatic properties to NB cells.

It could also be argued that the time before sacrifice of animals bearing NB8-CXCR4-C3 tumours after only 53 days, was too short and not sufficient to allow metastases to develop. This appeared unlikely, since macroscopic liver metastases were observed in the N91-E2 group already after 49 days. Furthermore, based on our experience in this model, bone marrow metastases usually occur within 50 days after tumour engraftment [19].

We could also rule out that NB8 clones expressed too low levels of CXCR4 to induce invasion or metastasis, as the levels of expression were comparable to that of the PC3-CXCR4 cells, which show enhanced invasive properties.

In contrast, CXCR4 overexpression induced a remarkable enhancement of, *in vivo* tumour growth, representing the essential observation of this study. Both CXCR4 overexpressing cell lines NB8-CXCR4-C3 and N91-CXCR4-14 displayed a considerably faster growth compared to their respective controls. Even N91-CXCR4-14 cells which did not show enhanced *in vitro* growth, proliferate significantly faster *in vivo*, indicating that endogeneous CXCR4-mediated effects on growth can be further increased and are most likely dependent on the tumor environment. Interestingly, secondary growth was also enhanced: macroscopic liver metastases in the N91-CXCR4-14 were all larger than 500 mm^3^ whereas the N91-E2 metastases were smaller than 500 mm^3^. Such observation strengthens the hypothesis that CXCR4/CXCL12 axis participates in the promotion of in vivo NB cell proliferation, together with other micro environmental factors, which need to be identified.

The impressive influence of CXCR4 on *in vivo* NB growth was further supported by CXCR4 knock-down experiments. *In vivo*, tumour growth of N91-shRNA-CS1 and N91-shRNA-CS2 clones with reduced endogenous CXCR4 was almost totally abrogated compared to N91-pAB303 control cells. This result confirms a role not only for exogenous but also for endogeneous CXCR4 in NB cell growth.

Under normal culture conditions i.e. in the presence of 10% FCS the *in vitro* proliferation assays showed a moderately increased growth of 2/3 NB8-CXCR4 clones compared to mock transduced cells. This discrete growth promoting effect may be ligand independent, considering that serum contains low levels of CXCL12, or be a result of an autocrine signal from low amounts of CXC12 produced by NB cells, as reported [22]. However, the NB8-C3 clone, displaying a moderate *in vitro* and a strongly enhanced *in vivo* growth, showed an increased *in vitro* growth in response to CXC12, indicating that CXCL12/CXCR4 axis can promote NB cell growth in vitro.

Moreover, NB8-CXCR4 cells were less sensitive to reduced serum concentration (2% FCS). This finding indicates that CXCR4 can, at least in part, compensate for serum depletion, and increases survival in stress conditions, a hallmark of malignancy. In NB8-CXCR4 cells CXCL12/CXCR4 activates ERK1/2, with oncogenic and tumour-promoting activities, consistent with the report by Hatse et al showing that endogenous CXCL12/CXCR4 signalling axis is critical for neuroblastoma cell survival and proliferation [42]. The pro-survival effect of CXCR4 observed in stress conditions may be due to the low amounts of CXCL12 produced by NB cells. The in vitro effect of CXCR4 on proliferation under reduced serum concentration was not observed for the IGR-N91 cell line, which may be due to the fact that the constitutive expression of CXCR4 in this cell line is already be sufficient to confer *in vitro* survival advantages. Indeed, both N91-shRNA-CS1 and N91-shRNA-CS2 CXCR4 silenced clones showed reduced cell proliferation *in vitro* in 10% serum and in reduced serum concentration.

Globally, our results show that exogeneous CXCR4 overexpression in IGR-N91 does not further increase their *in vitro* growth, migration and invasive properties, indicating that a subtle threshold of endogenous CXCR4 level necessary and sufficient for mediating growth, survival and migration is reached in these cells. In contrast, their *in vivo* growth could be further enhanced, probably by synergic signals provided by the tumour microenvironment.

The difference observed between the CXCR4 growth-promoting effects *in vitro* and *in vivo* indeed suggests that CXCR4 participates to a more complex growth-promoting phenomenon involving the tumour microenvironment. The high levels of CXCL12 found in the primary tumour site (adrenal gland) and in a major site of metastasis in our model (the liver) support the notion that it may confer survival and proliferative advantages to the NB cells and is consistent with a critical role of tumour microenvironment in primary and secondary tumour growth. In addition to a possible paracrine effect on tumour growth, the high levels of CXCL12 measured in the primary site (adrenal gland), represents a unique situation to NB where CXCL12 in the adrenal gland could also serve to retain the malignant cells in the primary site, rather than encourage them to metastasise [9]. Nevertheless, some tumour cells, possibly equipped with unidentified particularly invasive characteristics, do escape the primary tumour and may then use the CXCR4/CXCL12 axis to specifically migrate toward CXCL12 producing organs such as the liver. Thus the large size of liver metastases observed in mice engrafted with N91-CXCR4-14 cells, also supports a CXCR4-mediated enhanced growth at secondary sites as at primary organs.

In conclusion, this *in vivo* and *in vitro* study reveals unique growth-promoting effect of the CXCR4/CXCL12 axis in NB, demonstrated by concordant CXCR4 gain and loss of function assays. This study also emphasizes the importance to study tumour growth and progression in orthotopic conditions. The role of the microenvironment in the CXCR4-mediated growth and the underlying explanation for the lack of increased metastatic spread can only be identified using an orthotopic model [19]. In particular, the differential expression levels of CXCL12 ligand in primary and secondary target sites clarifies the role of the CXCR4/CXCL12 system in NB progression: it strongly enhances cell growth without increasing *in vivo* invasion.

Further *in vitro* molecular investigations will be needed to identify the growth and survival pathways involved in such CXCR4/CXCL12 responses. In particular, while some CXCR4-overexpressing related proteins have been already reported, a global CXCR4-mediated expression profile will be identified [43]. Key molecules of the CXCR4 signalling pathway could be new targets for specific NB therapeutic tools.

## Supporting Information

Figure S1 Zymographic assay of MMP-2 activity in NB cells culture media. Equal cell number (0.8×105) of different cell lines was plated in SFM. After 12 hours starvation, medium was collected and matrix metalloproteinase MMP-2, and MMP-9 activities were evaluated by gelatine zymography as described [44]. Gels were stained with Coomassie blue to check for equal sample loading. MMP-2 activity is represented as a band of gelatinolysis at 72 KDa. MMP-9 activity was not detected (not shown).
(0.14 MB TIF)
