# Supplementary figures and images for: The Chemokine Receptor CXCR4 Strongly Promotes Neuroblastoma Primary Tumour and Metastatic Growth, but not Invasion
Source: PLoS One. 2007 Oct 10;2(10):e1016. doi: 10.1371/journal.pone.0001016 (PMC1995764; doi:10.1371/journal.pone.0001016)

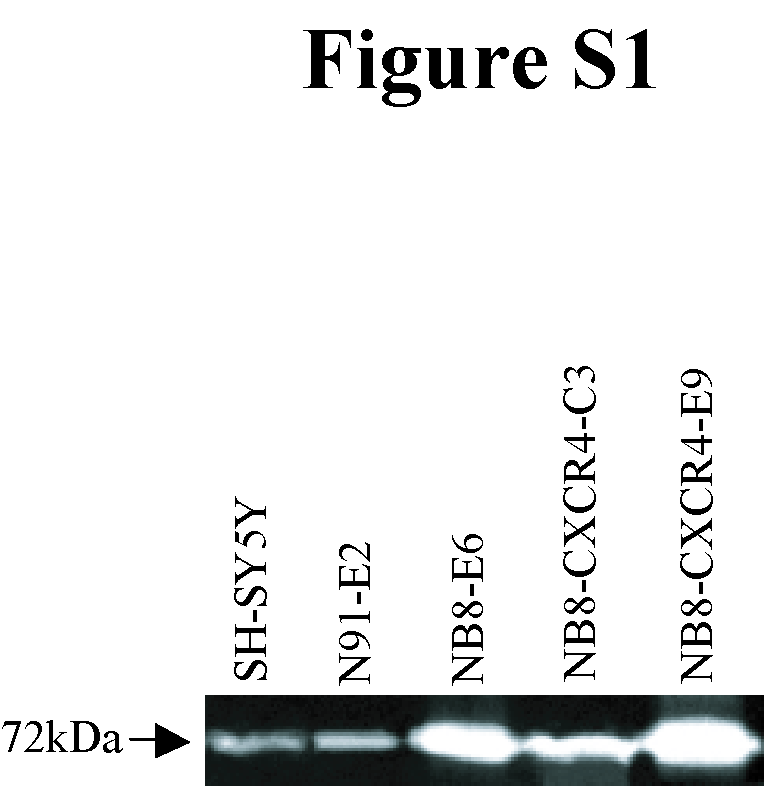

Supplement: Figure S1 — Zymographic assay of MMP-2 activity in NB cells culture media. Equal cell number (0.8×10^5^) of different cell lines was plated in SFM. After 12 hours starvation, medium was collected and matrix metalloproteinase MMP-2, and MMP-9 activities were evaluated by gelatine zymography as described [44]. Gels were stained with Coomassie blue to check for equal sample loading. MMP-2 activity is represented as a band of gelatinolysis at 72 KDa. MMP-9 activity was not detected (not shown). (0.14 MB TIF) [file pone.0001016.s001.tif]
